# Insight into the pathogenesis and nature of Central giant cell lesions of the jaws

**DOI:** 10.4317/medoral.20499

**Published:** 2015-02-14

**Authors:** Paul-Charles Edwards

**Affiliations:** 1 MSc, DDS, FRCD(C), Diplomate, American Board of Oral and Maxillofacial Pathology, Professor Dept. of Oral Pathology, Medicine and Radiology Indiana University School of Dentistry

## Abstract

Central giant cell lesions of the jaws are not uncommon. While the majority of these represent single, sporadic lesions, histologically identical lesions are seen in association with a number of other bone lesions, as well as in certain syndromes. This manuscript offers a brief update on recent developments in this area that provide new insight into the pathogenesis and nature of Central Giant Cell Lesions of the Jaws.

** Key words:**Central giant cell lesion, RASopathy

## Introduction

Central giant cell containing lesions of the jaws represent a heterogeneous group of relatively uncommon lesions, the protypical member of this group being the Central Giant Cell Lesion (CGCL).

These jaw lesions were previously classified under the rubric “giant cell variant of osteitis fibrosa”, or in the case of aggressive variants, characterized by one or more of extensive destruction, recurrence, loose teeth, cortical perforation, root resorption and/or, paresthesia, were regarded as a gnathic variant of the Giant Cell Tumor (GCT) of long bones. In 1953, Jaffe (1), introduced the term “giant cell reparative granuloma”, to convey both the presumed non-neoplastic nature of this process and to distinguish it from the GCT of extragnathic skeleton.

Among the many unanswered questions with respect to CGCLs of the jaws, arguably two of the most widely contemplated are: 1) defining the relationship between the spectrum of conditions that present with histologically indistinguishable lesions, and 2) the relationship between CGCLs of the jaw and extragnathic GCT.

## Pathogenesis of CGCL-like lesions in the spectrum of conditions presenting with histologically indistinguishable lesions

Lesions histologically indistinguishable from sporadic CGCLs of the jaws are seen in peripheral gnathic locations (peripheral giant cell granuloma (2,3), and in association with hyperparathyroidism (“brown tumor” of hyperparathyroidism). CGCL-like areas are also described in a greater than expected prevalence in association with other cent rally-occur ring jaw lesions, principally central odontogenic fibroma (4,5).

CGCL-like lesions of the jaws are the hallmark of cherubism, an autosomal dominantly inherited condition caused by mutations in the Sh3bp2 gene (6). Sh3bp2 codes for SH3-domain binding protein 2 (3BP2), a cytoplasmic adaptor protein that positively regulates transcriptional activity. Specifically, 3BP2 turnover is regulated by tankyrase-mediated poly(ADP-ribosyl)ation, which targets it to the ubiquitin-proteasome complex for degradation. In cherubism, mutated 3BP2 is uncoupled from protein destruction, resulting in 3BP2 stabilization and increased activation of SRC, SYK, and VAV signaling pathways, driving the osteoclast differentiation that is characteristic of cherubism. However, studies have, for the most part, failed to identify cherubism-associated Sh3bp2 mutations in sporadic CGCL cases (7,8).

A greater than anticipated prevalence of CGCL-like jaw lesions has also been observed in patients presenting with a number of syndromes. While not a universal occurrence, CGCL-like lesions of the jaws, often multiple in presentation (a rare occurrence in sporadic cases of CGCL, the vast majority of which present as a single lesion) have been reported in patients with Noonan syndrome (9,10); caused by multiple gene defects including PTPN11, KRAS, NRAS, SOS1, RAF1, BRAF, MAP2K1, SHOC2, CBL), neurofibromatosis type I (11,12); caused by NF1 mutations), cardiofacio-cutaneous syndrome; caused by multiple gene defects including BRAF, MAP2K1, MAP2K2, and KRAS), and Noonan syndrome with multiple lentigines (also known as LEOPARD syndrome; associated with gene defects including PTPN11, RAF1, and BRAF). Arguing against a direct genotype-phenotype association is the finding that a diverse group of syndrome-associated gene mutations have been identified in syndromic patients presenting with CGCL-like lesions of the jaws; including PTPN11, SOS1, BRAF, and MEK1 in Noonan Syndrome (13), BRAF and MEK1 in cardiofacio-cutaneous syndrome (13), and PTPN11 in Noonan syndrome with multiple lentigines (14). These syndromes have in common that they are characterized by overlapping facial and skeletal features and are caused by mutations at different points along the Ras/MAPK (mitogen-activated protein kinase) pathway, resulting in dysregulation of Ras/MAPK signaling. As a result, these different conditions are now grouped together under the category of “RAS/MAPK syndromes”; or “RASopathies”. While the exact pathogenesis linking alteration in the Ras/MAPK signal transduction pathway to the development of CGCL-like lesions in only a small subset of affected individuals remains to be determined, the RAS/MAPK pathway appears to be important in bone homeostasis.

Affected skeletal changes common to the RASopathies include include short stature, osteoporosis, chest wall deformities, scoliosis, and generalized increased bone resorption (15). In aggregate then, these findings suggest that CGCL-associated lesions of the jaws represent a diverse group of lesions in which osteoclastic overactivity is the hallmark feature which, in conjunction with as of yet unidentified precipitating factors; possibly to include trauma and vascular compromise, in predisposes to osteoclastic fusion a susceptible individual. The question remains as to what additional factors in the bone micro environment are involved in tipping the balance from physiologically altered but otherwise histologically normal appearing bone to outright CGCL development.

## Relationship between CGCLs of the jaw and extragnathic GCT

Discussions as to whether all aggressive CGCL lesions of the jaw are within the spectrum of CGCL (16) or whether subsets represent agnathic variant of the GCT of the long bone persist (17,18).

Behjati and colleagues (19) identified somatic mutations in the H3F3A gene, one of two genes coding for histone H3.3, in their patients with extragnathic GCTs. These mutations, reportedly restricted to the stromal cell population, led to amino acid substitutions of glycine 34 to tryptophan (p.Gly34Trp) or leucine (p.Gly34Leu) in 49 of 53 giant cell tumors of bone examined. Interestingly, the same authors reported p.Lys36Met alterations in 73 of 77 chondroblastomas; 5 involving the H3F3A gene and 68 involving H3F3B, the second gene encoding for histone H3.3.

While the mechanism by which these mutations predispose to GCT of extragnathic sites remains undefined, insight into the role of similar H3F3A mutations (p.Lys27Met, p.Gly34Arg and p.Gly34Val) in the development of glioma suggests a role for epigenetic transcriptional activation (20). Lys27Met-mutated gliomas are characterized by an almost complete absence of the trimethylated lysine residue H3K27me3 in all isoforms of histone H3, the main function of which is to repress transcriptional activity in chromatin. It follows, therefore, that the resultant loss of Lys27 methylation leads to the upregulation of multiple genes.

However, Gomes and colleagues (21) demonstrated that sporadic CGCL of the jaws do not share the H3F3A p.Gly34Trp or p.Gly34Leu mutations reported in GCT of long bones. These findings appear to add to the body of evidence that the CGCL of the gnathic bone is distinct and separate from the extragnathic GCT.

Interestingly, an additional recent study suggests that the relationship between H3F3A mutations and GCT pathogenesis may not be so clear. Kaneko and colleagues (22) were unable to identify the same H3F3A mutations described by Behjati and colleagues (19) in any of 20 Asian patients with GCT of the extragnathic skeleton. Instead, in majority of their GCT patients, they reported specific isocitrate dehydrogenase gene mutations.

## Conclusion

While significant progress continues in our quest to understand this enigmatic group of conditions, much remains to be defined with respect to the pathogenesis of CGCLs of gnathic bone.
